# On the definition and interpretation of voice selective activation in the temporal cortex

**DOI:** 10.3389/fnhum.2014.00499

**Published:** 2014-07-08

**Authors:** Anja Bethmann, André Brechmann

**Affiliations:** Special Lab Non-Invasive Brain Imaging, Leibniz Institute for NeurobiologyMagdeburg, Germany

**Keywords:** voice processing, speaker recognition, famous persons, anterior temporal lobes, superior temporal sulcus, auditory processing stream, selectivity, equivalence testing

## Abstract

Regions along the superior temporal sulci and in the anterior temporal lobes have been found to be involved in voice processing. It has even been argued that parts of the temporal cortices serve as voice-selective areas. Yet, evidence for voice-selective activation in the strict sense is still missing. The current fMRI study aimed at assessing the degree of voice-specific processing in different parts of the superior and middle temporal cortices. To this end, voices of famous persons were contrasted with widely different categories, which were sounds of animals and musical instruments. The argumentation was that only brain regions with statistically proven absence of activation by the control stimuli may be considered as candidates for voice-selective areas. Neural activity was found to be stronger in response to human voices in all analyzed parts of the temporal lobes except for the middle and posterior STG. More importantly, the activation differences between voices and the other environmental sounds increased continuously from the mid-posterior STG to the anterior MTG. Here, only voices but not the control stimuli excited an increase of the BOLD response above a resting baseline level. The findings are discussed with reference to the function of the anterior temporal lobes in person recognition and the general question on how to define selectivity of brain regions for a specific class of stimuli or tasks. In addition, our results corroborate recent assumptions about the hierarchical organization of auditory processing building on a processing stream from the primary auditory cortices to anterior portions of the temporal lobes.

## 1. Introduction

fMRI studies revealed that the temporal cortices of humans are activated during the perception of other persons' voices. Especially areas along the superior temporal sulci (STS) and in the anterior temporal lobes (ATLs) have been argued to be directly involved in processes concerned with the analysis and recognition of human voices. The relevance of areas along the STS has been emphasized by experiments that compared voice perception to the processing of other meaningful environmental sounds. Particularly areas in the upper bank of the STS indicated a strong preference for voices when those comparisons were made (Belin et al., 2000, 2002; Fecteau et al., 2004). In contrast, designs that controlled for speech recognition processes often identified the ATLs as voice-specific areas. This was achieved by presenting non-speech vocalizations (Belin et al., 2002; Meyer et al., 2005), by directly contrasting voice and speech processing (Belin and Zatorre, 2003; von Kriegstein et al., 2003), or by comparing the processing of familiar and unfamiliar voices (Birkett et al., 2007; Bethmann et al., 2012).

**Figure 1:**
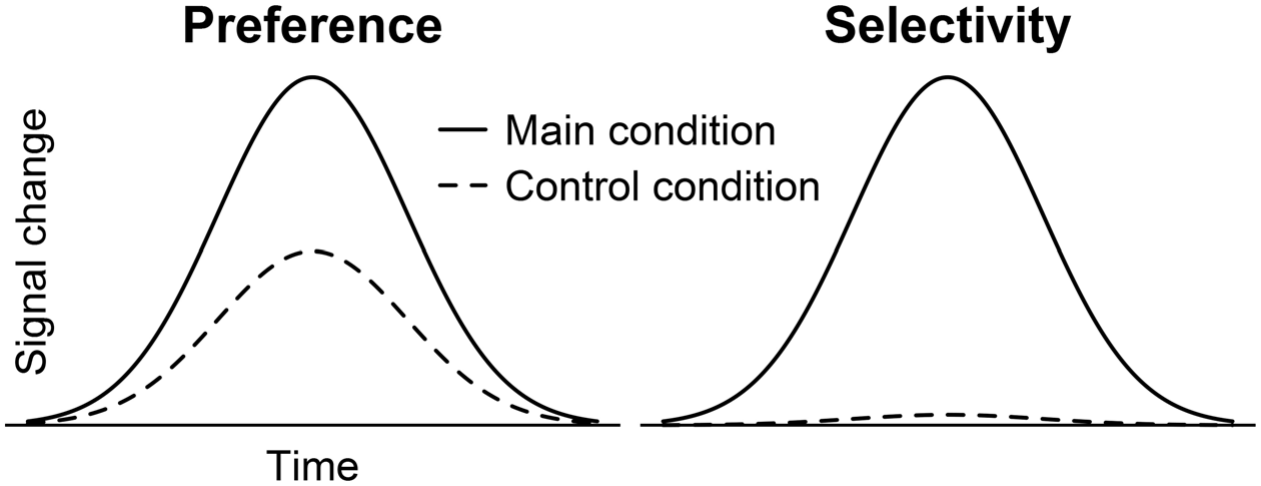
**BOLD time courses showing preferential vs. selective activation depending on the amplitude elicited by a control condition**. Provided the main condition induced the larger amplitude, the control condition could evoke significant activation as well (preference) or fail to raise the activation above a resting baseline level (selectivity).

Based on these findings, the suggestion has been put forward that parts of the temporal lobes serve as “voice-selective areas” (Belin et al., 2000, 2002). In our view, however, this conclusion is too strong. Previous data often showed a preference for voices over other sounds (Belin et al., 2002; Fecteau et al., 2004) but seldom true selectivity in the strict sense where control stimuli or tasks evoked no activation (Figure 1). Only one paper that we are aware of argued to have found such a strict voice selectivity (von Kriegstein et al., 2003). The authors, however, missed to statistically test the absence of activation in response to the control stimuli. Yet, such a demonstration is absolutely required because non-significant activation does not confirm the absence of activation.

Faces (Kanwisher et al., 1999), body parts (Downing et al., 2001), scenes (Epstein and Kanwisher, 1998), language (Fedorenko et al., 2011), and letters (Cohen and Dehaene, 2004) are further examples of stimuli that were claimed to be selectively processed by a specific brain region. But similar to voice selectivity, preferential rather than selective activation was found.

The idea behind the search for selective brain responses is to identify cognitive modules (Fodor, 1983) and to link them to specific brain regions. An important property of modules is their domain specific application, which means that they should only respond to stimuli of a particular category (Coltheart, 1999, 2011). Coltheart (1999, p. 118) states that “to say that there is a domain-specific face recognition module is to say that there is a cognitive system that responds when its input is a face, but does not respond when its input is, say, a written word, or a visually-presented object, or someone's voice.” Hence, in order to detect brain areas that operate in a domain specific manner, selective activation should be preferred over preferential activation (Joseph et al., 2006; Pernet et al., 2007).

Since the issue of the selective bias of brain regions toward particular stimuli is still unresolved, the aim of the present study was to analyze whether regions exist in the human brain that selectively respond to a particular stimulus type. The stimuli used here were human voices and we examined whether any area along the STS or in the ATLs reveals voice-selective activation in the strict sense with a statistically proven absence of activation in response to control stimuli. These were sounds of animals and musical instruments because of their wide differences compared to human voices (e.g., in their acoustic, semantic, and linguistic content). In previous studies, these comparisons evoked preferential activation patterns (Belin et al., 2000, 2002; Fecteau et al., 2004). But we claim that any potential voice-selective area should show selective activation to voices when compared to control stimuli. If this prerequisite is not fulfilled, a brain region should not be termed voice-selective.

## 2. Materials and methods

### 2.1. Participants

A total of 12 young adults (8 women) at the age of 25.3 ± 3.2 years (mean ± *SD*) who were native German speakers participated in the present study on a voluntary basis. None of the participants reported any history or evidence of neurological, psychiatric, or audiological symptoms. All gave written informed consent according to local institutional guidelines and were paid a small hourly stipend. The study received prior approval by the ethics committee of the Otto-von-Guericke University Magdeburg, Germany. The current version of the World Medical Association's Declaration of Helsinki (http://www.wma.net) was respected.

### 2.2. Stimuli

Three kinds of auditory stimuli (90 stimuli in total, 44,100 Hz, 16 bit, mono, normalized to 100%, fade-in and fade-out period of 30 ms) were presented binaurally. The first group of stimuli were utterances spoken spontaneously by 20 famous (Angela Merkel, Thomas Gottschalk; 8 female) and 10 unknown (4 female) German people. All speakers were adults between 20 and 80 years of age. A higher number of famous than unknown voices was chosen to balance the number of familiar and unfamiliar speakers because previous studies have demonstrated that voice recognition is rather low (Hanley and Damjanovic, 2009; Bethmann et al., 2012). The utterances had a duration of 2 s and consisted of several consecutive words forming short phrases. The excerpts were chosen such that the content gave no hint as to the identity of the speaker (Bethmann et al., 2012). The utterances were extracted from video clips published on the websites of public German broadcasting corporations. The second stimulus group consisted of 20 characteristic animal sounds (dog, sheep, frog) and 10 sounds that were ambiguous with regard to the animal that might have produced that sound. The third group consisted of 20 sounds and melodies produced by musical instruments (piano, guitar, saxophone) and 10 sounds and melodies that were of synthetic origin and not attributable to a particular instrument. The animal and musical sounds, which also had a duration of 2 s, were downloaded from various websites that allowed to play the sounds for free. All sounds were recorded and processed using the software Cool Edit 2000 (Syntrillium Software Corporation, Phoenix, USA).

Sound properties were analyzed by the computer program Praat, version 5.3.71 (Boersma and Weenink, 2014). The mean intensities did not vary significantly between the stimulus categories but several other acoustic parameters did (Mann-Whitney-U test, two-tailed, see Table 1). Most importantly, the fundamental frequencies (F0) were lower and less variable in human voices than in the other stimuli. Significant differences also applied to the parameters jitter, shimmer, and to the harmonics-to-noise ratio, where human voices fell between the animal and musical sounds.

**Table 1:** **Acoustic properties of the three stimulus categories**.

**Property**	**Human voices**	**Animal sounds**	**Sounds of musical instruments**	***p***
Intensity mean (db)	76.3	73.5	76.8	0.156
F0 mean (Hz)	168.6^*^	301.9	246.5	0.001
F0 SD (Hz)	35.5^*^	62.6	61.2	0.001
Jitter local %	2.8	4.1	2.3	0.015
Shimmer local %	12.4	15.9	11.7	0.010
Harmonics-to-noise ratio (db)	9.5	6.0	12.9	0.003

**Asterisks:** *mark those acoustic properties by which human voices differed unidirectionally both from animal sounds and musical instruments*.

Hence, human voices differed from sounds of animals and musical instruments in various ways, e.g., in their acoustic properties (fundamental frequencies), their linguistic content (carried by human voices but not by animal sounds and musical instruments) or their specificity of semantic processing (individual level processing for persons and basic level processing for animals and musical instruments). We reasoned that only those brain areas should be considered as candidates for “voice-selective areas” if they respond in a strictly voice-selective manner when compared to the largely different stimulus types that were used here.

### 2.3. Experimental designs

Each sound was presented only once in a pseudo-randomized order (with regard to category and familiarity) using a slow event-related fMRI design with a rest period (with continuing scanner noise) of 12 s after each presentation.

The voices served as main condition whereas animals sounds and musical instruments acted as control conditions. In order to evoke semantic and not only acoustic processing, the participants' attention was directed to the origin of the sounds (i.e., the speakers, the animal species or types of musical instruments) by using a combined familiarity and naming task. For this reason, familiar and unfamiliar/ambiguous stimuli were used. The participants were asked to indicate whether they had identified the speakers, animals, or instruments to a degree that they were able to name the stimuli at the individual level in the case of human voices and at the basic level in the case of animals and musical instruments. The subjects' response consisted of pressing one of two buttons (index finger = name retrieval, middle finger = no name retrieval).

### 2.4. Imaging methods

#### 2.4.1. Data acquisition

Magnetic resonance imaging was conducted at a 3T Siemens Trio scanner (Siemens, Erlangen, Germany) using a head array receive coil with eight channels. Stimulus presentation was timed by the software Presentation 9.20 (Neurobehavioral Systems, Inc., Albany, USA), which was also used to register the manual responses via buttons. The stimuli were presented through MR-compatible headphones adjusted to a comfortable listening level (MR confon, Magdeburg, Germany). The participants were requested to keep their eyes closed during all scans.

In each subject, three scan sequences were performed. At first, high-resolution T_1_-weighted images with 1 mm isotropic resolution were acquired using an MPRAGE sequence (192 gapless axially oriented slices, field of view = 256 × 256 mm, *TR* = 2500 ms, *TE* = 4.77 ms, *TI* = 1100 ms). The scan covered the whole brain and served to reconstruct the individual three-dimensional brain anatomy. Secondly, a T_1_-weighted, anatomical, two-dimensional data set was acquired with an IR-EPI sequence (*TR* = 20,000 ms, *TE* = 34 ms, *TI* = 1450 ms). Other parameters as orientation and geometry were equal to the functional scans which were done in a last step. The functional images were taken using a T^*^_2_-weighted GE-EPI sequence (32 axially oriented slices, voxel size = 3 × 3 × 3 mm, interslice gap = 0.3 mm, field of view = 192 × 192 mm, matrix = 64 × 64 voxels, *TR* = 2000 ms, *TE* = 30 ms, *TI* = 62 ms, flip angle = 80°). The latter image sets were oriented roughly parallel to the sylvian fissure with only minor differences between the subjects to ensure maximal coverage of the entire cerebrum, excluding only the most superior frontoparietal regions and parts of the occipital lobes.

The functional images were acquired in a single run that lasted 21 min 28 s and resulted in 644 volumes. Each examination took less than 1 h.

#### 2.4.2. Data preprocessing

All processing steps and the analysis of the MRI data were done using the BrainVoyager QX software, version 1.10.4 (Brain Innovation, Maastricht, The Netherlands). The anatomical 3D data were transformed into AC-PC and Talairach standard space Talairach and Tournoux (1988). After having imported the functional data, a standard sequence of preprocessing steps was applied, including slice scan time correction, head motion correction, linear trend removal, and temporal highpass filtering with two cycles per scan. No spatial smoothing was utilized. Finally, the functional data were combined with the anatomical data sets to display activations in 3D space.

Additionally, the functional data were inspected thoroughly for severe signal intensity (gray level) fluctuations resulting from head motion. For this purpose, the automated head motion correction procedure, which resulted in estimated translation and rotation parameters for each spatial direction, was analyzed. In particular, the data sets were checked for jerky movements as these can lead to signal artifacts. A jerky move was defined as a translation or rotation of the head from one volume to the next in the magnitude of 0.5 mm or 0.5° in one spatial direction or of 1.0 as the sum of all directions. The respective volumes were eliminated to correct for outliers.

### 2.5. Analysis

#### 2.5.1. Activation maps

Parametric activation maps were generated by applying a univariate general linear model (GLM) to each voxel. The model was convolved with the two gamma hemodynamic response function using the standard parameters in BrainVoyager QX 2.8.0. Predictor variables of the estimated time course were the three conditions of the experiment (H, human voices; A, animal sounds; M, musical instruments) but also the individual head motion parameters that had been identified with the head motion detection procedure. These were included to weight and reduce the influence of smaller head shifts on the signal change.

A multi-subject GLM was calculated using a random effects analysis. Two activation maps were generated at an FDR (false discovery rate) corrected significance level of *q*_FDR_ < 0.05 (Benjamini and Hochberg, 1995; Genovese et al., 2002). Voxels were included only if they formed a cluster of at least four contiguous voxels (each having a size of 3 × 3 × 3 mm). The first map (I) highlighted those voxels that were significantly [*t*_(11)_ ≥ 2.50] more activated by human voices than during rest (*H*^+^). The analyzed brain region was bounded by a masking procedure to the temporal lobes. The size of the mask was defined according to the region of interest approach described below in section *ROI analysis*. For the second map (II), the analyzed brain region was bounded by a mask to those voxels that were significantly activated in the first map to ensure that only those voxels were included that were significantly activated by human voices. In addition, the second map required that human voices resulted in a significantly [*t*_(11)_ ≥ 2.42] stronger neural response than animals or musical instruments. More precisely, the contrast revealed those voxels whose activation to voices when multiplied by two was significantly higher than the sum of the activation to animals and musical instruments (2*H* > *A* +*M*). Hence, the second map showed voxels that were significantly activated by human voices and, in addition, more strongly activated by voices than by animals or musical instruments (conjunction of *H*^+^ and 2*H* > *A* + *M*).

Subsequently, each voxel in the temporal lobes that was significantly activated by human voices compared to rest (*H*^+^) was analyzed for its specificity regarding voice processing. Five specificity patterns were defined by varying the significance level and the contrast settings between the three conditions. An FDR corrected significance level of *q*_FDR_ < 0.05 was used when real significance was required. The level was set to *p* ≤ 0.2 to identify voxels without significant activation. For example, an area significantly activated by condition A but not B was found by requiring that A caused significant activation (*q*_FDR_ < 0.05) and by reducing the resulting voxels by those that were activated even to the slightest degree by B (*p* ≤ 0.2).

The five specificity patterns were defined in the order 5, 1, 4, 3, 2 with higher numbers indicating higher specificity. For pattern 5 (*strong selectivity*), those voxels were identified that were significantly more activated by human voices than by animals or musical instruments and that revealed a significant deflation of the BOLD signal from animal sounds and musical instruments compared to rest (*H*^+^, *H* > *A, H* > *M, A*^−^, *M*^−^, each *q*_FDR_ < 0.05). The requirement of a signal decrease was introduced to ensure that there was definitely no activation to animals and musical instruments above baseline, which cannot be inferred from non-significant activation (see section Statistics). For pattern 4 (*selectivity*), voxels were determined that showed a preference for human voices and that neither were activated by animals or musical instruments nor belonged to the voxels of pattern 5 (*H*^+^, *H* > *A, H* > *M*, each *q*_FDR_ < 0.05; without voxels showing *A*^+^ or *M*^+^, both *p* < 0.2, and without voxels belonging to pattern 5). Pattern 3 (*preference*) was composed of voxels that showed a preference for voices over animals and musical instruments but did not belong to any of the other patterns (*H*^+^, *q*_FDR_ < 0.05; *H* > *A, H* > *M*, both *p* < 0.2; without voxels belonging to pattern 1, 4, 5). Pattern 1 (*equivalence*) comprised those voxels that were significantly activated both by human voices and sounds of animals and musical instruments but did not involve voxels that showed even the slightest preferences for voices (*H*^+^, *A*^+^, *M*^+^, each *q*_FDR_ < 0.05; without voxels showing *H* > *A* or *H* > *M*, both *p* < 0.200). Finally, pattern 2 (*remainder*) consisted of those voxels that were significantly activated by voices but did not fall into one of the other patterns (*H*^+^, *q*_FDR_ < 0.05; without voxels belonging to pattern 1, 3, 4, 5).

In addition to the multi-subject analysis, activation maps were generated in each participant separately by applying a single-subject GLM. The maps required that the BOLD response was higher in response to human voices than during rest (H^+^). Hence, those areas were identified that were generally involved in voice processing. Of the resulting voxels, only those were analyzed that were activated at a significance level of *t*_(634)_ ≥ 4.00 (*q*_FDR_ < 0.001) and that formed a cluster of at least four adjacent voxels. Only those voxels were considered whose first functional EPI signal had an intensity (gray level) of at least 75 (range: 0–225; 0 = low intensity, tissue with slow relaxation of magnetization). This was done to reduce the number of multiple comparisons and to minimize signal artifacts from brain areas with low signal intensity. The latter was especially important because the anterior temporal lobes, which were mainly addressed by the present study, are in very close proximity to tissue transitions. The procedure allowed to omit the outermost voxels of the temporal brain structures from the analysis, which are particularly prone to signal artifacts. The activation maps of the different subjects were evaluated separately by means of individually defined regions of interest.

#### 2.5.2. ROI analysis

Two regions of interest (ROIs) were created to measure the extent of activation in the left and right temporal lobe. These ROIs were created individually in each participant using their structural MRI data (Bethmann et al., 2012). The position of the ROIs was aligned to the slope of the STS along the y-axis of the brain from anterior to posterior coordinates. As the slope of the STS alters from lateral to more medial slices, the position of the ROIs was adjusted accordingly. Nine consecutive sagittal ROI slices each had an identical slope, with four voxels left and right of *x* = ±65, ±56, ±47, and ±38 (Talairach coordinates). This was done separately for each hemisphere. Overall, the ROIs extended from lateral *x* = ±69 to medial *x* = ±34.

**Figure 2:**
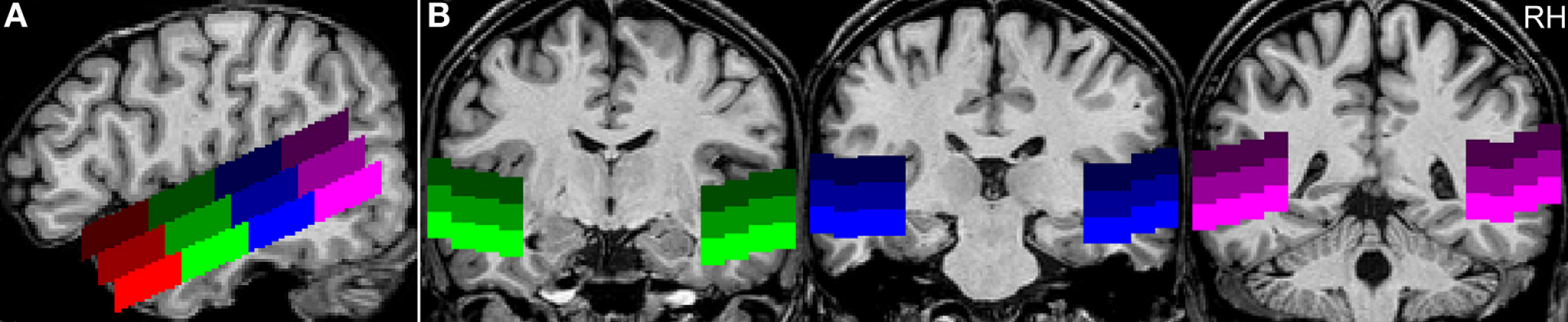
**Example for the positioning of the regions of interest**. **(A)** The sagittal view of a single subject's brain shows the position of the ROIs along the superior temporal sulcus at Talairach coordinate *x* = 50 (RH, right hemisphere). The upper row of ROIs covers the superior part of the STG (sSTG), the middle row the inferior part of the STG (iSTG), and the lower row the superior part of the MTG (sMTG). Red ROIs are located in the anterior temporal lobe (a), green ROIs in the mid-anterior part (ma), blue ROIs in the mid-posterior part (mp), and purple ROIs in the posterior temporal lobe (p). **(B)** Coronal views at *y* = −10 (green), −25 (blue), and −41 (purple).

The ROIs were bilaterally subdivided into 12 subregions with three rows and four columns (Figure 2). Each row had a height of ten voxels (i.e., 10 mm). The upper row covered the superior part of the STG (*sSTG*), the middle row the inferior part of the STG (*iSTG*), and the lower row the superior part of the MTG (*sMTG*). Each row was further subdivided into four ROIs; an anterior ROI (*a*), a mid-anterior ROI (*ma*), a mid-posterior ROI (*mp*), and a posterior ROI (*p*). The upper row ran from anterior *y* = 29 to posterior *y* = −50 (*a* with 29 ≥ *y* ≥ 10; *ma* with 9 ≥ *y* ≥ −10; *mp* with −11 ≥ *y* ≥ −30; *p* with −31 ≥ *y* ≥ −50). Compared to the upper row, the middle row was moved backwards by five voxels, the lower row by 10 voxels. Each of the 24 ROIs was composed of 7200 voxels (voxel size = 1 mm^3^). Their centers are given in Table 2.

**Table 2:** **Position of the regions of interest in Talairach space**.

	**a**		**ma**		**mp**		**p**	
	**LH**	**RH**	**LH**	**RH**	**LH**	**RH**	**LH**	**RH**
sSTG	20; −13	20; −13	−01; −01	−01; −01	−20; 10	−20; 11	−40; 22	−40; 24
iSTG	14; −20	14; −20	−06; −08	−06; −08	−26; 03	−26; 04	−46; 15	−46; 17
sMTG	10; −27	10; −27	−10; −15	−10; −15	−30; −04	−30; −02	−50; 08	−50; 10

*Given are the means of their centers (y; z) across all subjects. The x-coordinate was always −52 for the left hemisphere and 52 for the right hemisphere. a, anterior; iSTG, inferior STG; LH, left hemisphere; ma, mid-anterior; mp, mid-posterior; p, posterior; RH, right hemisphere; sMTG, superior MTG; sSTG, superior STG*.

From each of the 24 ROIs, one BOLD value for each condition (see section BOLD signal time courses) and the number of significantly activated voxels (giving the activated volume) were extracted for further statistical analysis.

#### 2.5.3. BOLD signal time courses

The BOLD signal intensities were averaged over all trials of a condition starting 2 s before stimulus presentation and ending 16 s after stimulus onset. The baseline activation level was determined from three time points. These were the two time points just before stimulus presentation and the time point during presentation. Each time point had a duration of 2 s, which was the time to scan the whole brain once (= one brain volume). For the ROIs of the separate-subject analysis, one BOLD value for each condition was extracted from the resulting signal time course. For this, we chose the mean of the BOLD intensities from volume three to six after stimulus onset.

#### 2.5.4. Statistics

The statistical analyses of the behavioral and functional data were performed using the software package IBM SPSS Statistics (IBM Corporation, New York, USA). The data were examined for normal distribution using the Kolmogorov-Smirnov test. Since the majority of data 93.3% were found to be normally distributed and since we were not aware of a statistical program offering a multi-factorial non-parametric repeated measurements ANOVA, we decided to apply parametric tests for within-subjects designs. Before running the analyses of variance, the data were analyzed with Mauchly's sphericity test. If the sphericity assumption was found to be violated, the Greenhouse-Geisser correction was used to produce a valid significance level. When an omnibus test was complemented by *post hoc* tests, the *p* values from the pairwise comparisons were adjusted applying the Bonferroni method to counteract an inflation of the familywise error rate (marked by *p*_*B*_, where *p*_*B*_ = *n* × *p*, with *n* being the number of comparisons). Statistical results were given for two-tailed testing. Raw scores were presented as mean ± standard error of the mean.

The separate-subject data were further examined for ROIs that were activated by voices but not by sounds of animals or musical instruments (strict voice selectivity). Since non-significant results do not verify the absence of activation when standard hypothesis testing is used (because of the lack of control over the type II error), the non-superiority variant of the equivalence test procedure was applied (Westlake, 1972; Blackwelder, 1982; Walker and Nowacki, 2011; Meyners, 2012). Equivalence testing involves the calculation of a confidence interval around the sample mean and the examination whether the confidence interval is within pre-specified boundaries of equivalence. The non-superiority variant requires the estimation of only the upper confidence limits and is suited to ask whether the activation intensity was lower than an upper confidence boundary. In the present study, right-sided 97.5% confidence limits were calculated and the confidence boundary was defined as 10% of the BOLD signal induced by voices. Hence, a BOLD response by animals and musical instruments lower than 10% of the BOLD signal of the human voices was accepted as null activation (with the type I error of α ≤ 0.025). This definition is similar to, but clearly more strict than the selectivity criterion by Spiridon and Kanwisher (2002), which only required that the BOLD response to the preferred stimuli was two times larger than that of the non-preferred control stimuli. In contrast, a ROI was recognized as a potential voice-selective area in the current study if the upper 97.5% confidence limit of the BOLD signal of the control conditions was lower than 10% of the BOLD signal of the voices.

## 3. Results

### 3.1. Behavioral data

The human voices (H), animal sounds (A) and musical instruments (M) had to be rated for stimulus familiarity/nameability during the scans. Each stimulus category consisted of 20 stimuli expected to be familiar to the participants and 10 stimuli expected to be unfamiliar. The behavioral data of 11 subjects were analyzed. The data of one participant was not used because the responses seemed not to correspond to the instructions. Indications were a very low number of recognized animals (*n* = 3) and a higher number of recognized persons (*n* = 10), which contrasted markedly with the pattern of the other subjects (see below).

The number of familiar stimuli varied significantly across categories [*F*_(2)_ = 55.1, *p* < 0.001] with the recognition of fewer persons than animals or musical instruments [*t*_(10)_ ≥ 7.7, *p*_*B*_ < 0.001]. The number of recognized animals and musical instruments did not differ significantly [*t*_(10)_ = 0.3, *p*_*B*_ = 0.999]. Also the response times differed between the stimulus categories [*F*_(2)_ = 11.6, *p* < 0.001] with longer response times to human voices than to musical instruments and these again to animal sounds (Table 3). However, *post-hoc* tests using the Bonferroni correction method revealed significant differences between the response times for human voices and animals only [*t*_(10)_ = 4.3, *p*_B_ = 0.004] but neither between human voices or animals vs. musical instruments [*t*_(10)_ ≤ 2.6, *p*_*B*_ ≥ 0.080]. Misses were very seldom with 1.6 ± 2.5 (mean ± standard deviation) human voices without response, 0.8 ± 1.3 animal sounds and 1.0 ± 1.5) musical instruments.

**Table 3:** **Behavioral data**.

	**Number of familiar stimuli**		**Response times in [s]**	
	**Mean**	***SD***	**Mean**	***SD***
Human voices	8.8	2.6	3.81	1.00
Animal sounds	16.5	2.5	3.12	0.75
Musical instruments	16.7	2.1	3.37	0.98

*Presented are the number of recognized items and the response times for the three stimulus types (SD, standard deviation)*.

### 3.2. Imaging results

#### 3.2.1. Multi-subject analysis

To get a general idea of the areas in the temporal lobes that are involved in human voice processing, a multi-subject GLM was calculated at an FDR corrected significance level of *q*_FDR_ < 0.05 using a random effects analysis.

**Figure 3:**
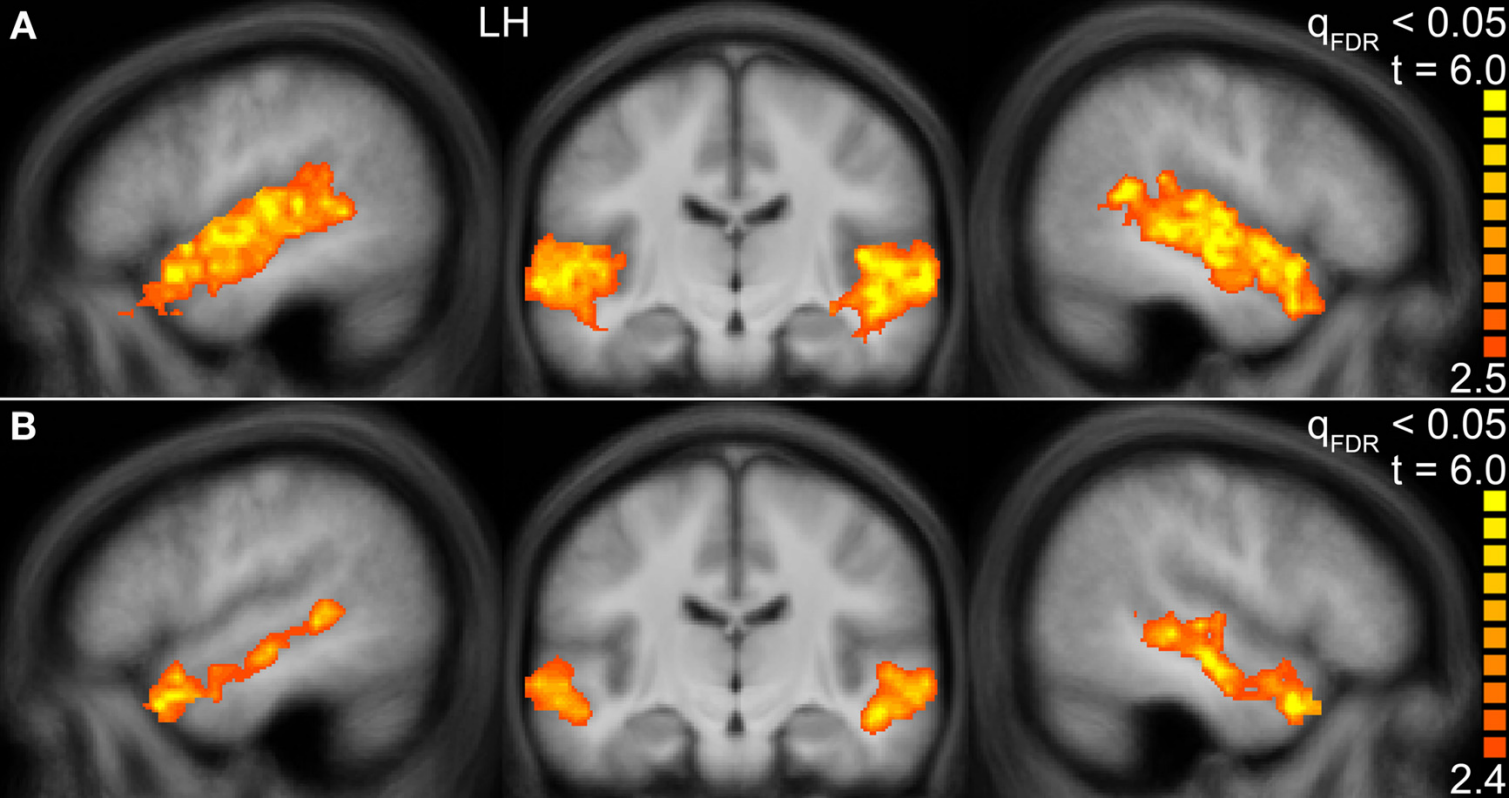
**Group activation maps of the temporal lobes at the FDR corrected significance level of *q*_FDR_ < 0.05 for the contrasts (A) Human voices > Rest (*H*^+^) and (B) Human voices > Animal sounds + Musical instruments (conjunction of *H*^+^ and 2*H* > *A* + *M*)**. The sagittal views are shown in Talairach space at *x* = −49 (LH, left hemisphere) and *x* = 47 (right hemisphere), the coronal views at *y* = −16.

**Figure 4:**
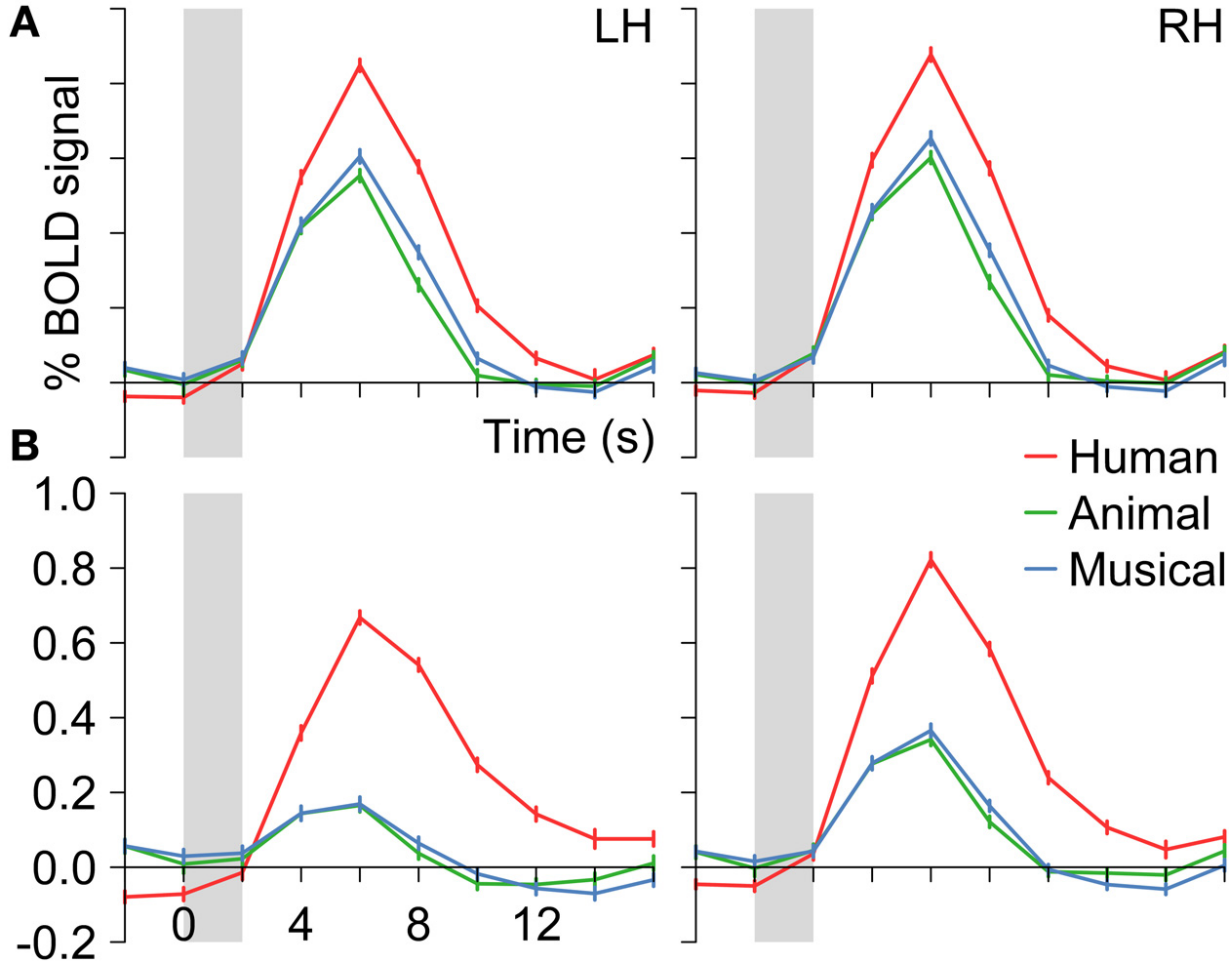
**Group BOLD time courses for the contrasts (A) Human voices > Rest (*H*^+^) and (B) Human voices > Animal sounds + Musical instruments (conjunction of *H*^+^ and 2*H* > *A* + *M*) averaged over the activated voxels in the temporal lobes (see Figure 3)**. Gray bars indicate the period of stimulus presentation.

First, we identified those voxels of the temporal lobes that were activated by human voices vs. rest (human voices > rest, H^+^). In this way, the whole STG of both hemispheres was found to be activated (Figure 3A). The activated volume had a size of about 31000 mm^3^ in each hemisphere (Table 4I). The corresponding BOLD (Figure 4A) signal shows that the maximum of the mean BOLD amplitude was approximately 0.9% for the voices (*t* ≥ 20.0, *p* < 0.001). The signal also revealed that animal sounds and musical instruments evoked strong and significant activation compared to rest as well (*t* ≥ 12.7, *p* < 0.001) even if the amplitude was slightly lower than for voices (by approximately 0.3%; *t* ≥ 5.5, *p* < 0.001).

**Table 4:** **Details for the regions of interest identified by the multi-subject analysis**.

	**ROI**	**Volume**	**Center**			**Human**		**Animal**		**Musical**		**H > A + M**	
			***x***	***y***	***z***	***t***	***p***	***t***	***p***	***t***	***p***	***t***	***p***
I	LH	31011	−51	−16	1	20.04	0.000	13.81	0.000	12.68	0.000	5.55	0.000
	RH	31904	50	−17	0	23.57	0.000	15.56	0.000	20.96	0.000	7.64	0.000
II	LH	12547	−54	−11	−7	15.00	0.000	2.95	0.013	2.84	0.016	9.59	0.000
	RH	12726	50	−11	−8	24.83	0.000	7.18	0.000	7.50	0.000	17.28	0.000
1	LH	71	−49	11	−22	2.61	0.024	−6.18	0.000	−6.21	0.000	5.24	0.000
	RH	69	46	9	−25	4.76	0.001	−4.91	0.000	−5.13	0.000	8.87	0.000
2	LH	4479	−53	−8	−10	7.27	0.000	−0.61	0.552	−0.52	0.613	8.34	0.000
	RH	2910	49	−4	−15	7.17	0.000	0.08	0.937	0.16	0.874	16.63	0.000
3	LH	10293	−55	−15	−1	20.13	0.000	7.38	0.000	6.41	0.000	7.56	0.000
	RH	12071	51	−16	−2	36.14	0.000	11.85	0.000	11.92	0.000	11.68	0.000
4	LH	7067	−48	−13	1	19.16	0.000	20.11	0.000	21.88	0.000	3.62	0.004
	RH	7748	51	−16	2	21.01	0.000	16.11	0.000	22.63	0.000	4.44	0.001
5	LH	9101	−49	−23	8	13.55	0.000	17.72	0.000	19.77	0.000	0.93	0.373
	RH	9106	49	−23	7	12.36	0.000	14.77	0.000	23.97	0.000	0.47	0.645

*(I) Human voices > Rest (H^+^), (II) Human voices > Animals sounds + Musical instruments (conjunction of H^+^ and 2H > A + M), 1, equivalence; 2, remainder; 3, preference; 4, selectivity; 5, strong selectivity. The activated volume in mm^3^, the Talairach coordinates of the gravity center, t values and significance levels for human voices (H), animal sounds (A), musical instruments (M), and the contrast H > A + M are given*.

The voxels identified before were then reduced to those that were significantly more strongly activated by voices than by sounds of animals or musical instruments [H^+^ ∧ (2*H* > *A* + *M*)]. This procedure revealed an activated volume of about 12500 mm^3^ in each hemisphere (Table 4II). Figure 3B shows that these voxels were mainly located around the STS of both hemispheres all along their horizontal lengths reaching deeply into the fundi of the sulci, whereas the superior part of the STG revealed only little activity. Although all categories evoked significant activation (*t* ≥ 2.8, *p* ≤ 0.016), the averaged BOLD amplitudes revealed large activation differences between the voices on the one hand (above 0.7%) and animals and musical instruments on the other hand (below 0.4%; *t* ≥ 9.6, *p* < 0.001; Figure 4B).

**Figure 5:**
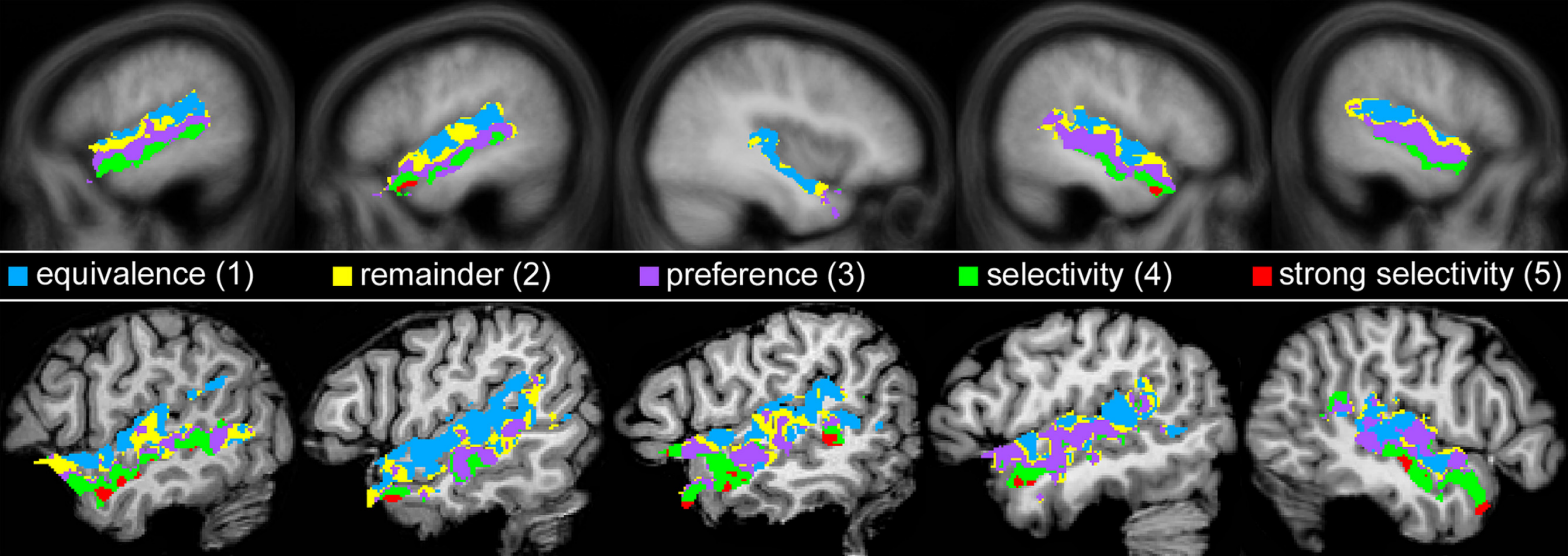
**Map of the areas in the temporal lobes that were significantly (*q*_FDR_ ≤ 0.05) activated by human voices (H^+^) with each color representing a different activation pattern regarding voice processing (Nos. 1–5)**. For details see section *Activation maps*. Figure 6 shows the corresponding BOLD time courses. Upper row: Multi-subject analysis of all 12 subjects. The sagittal views are shown in Talairach space at *x* = −53, −49, 37, 47, 52 (from left to right). Lower row: Five individual subjects at *x* = −52, −52, −52, −49, 48.

**Figure 6:**
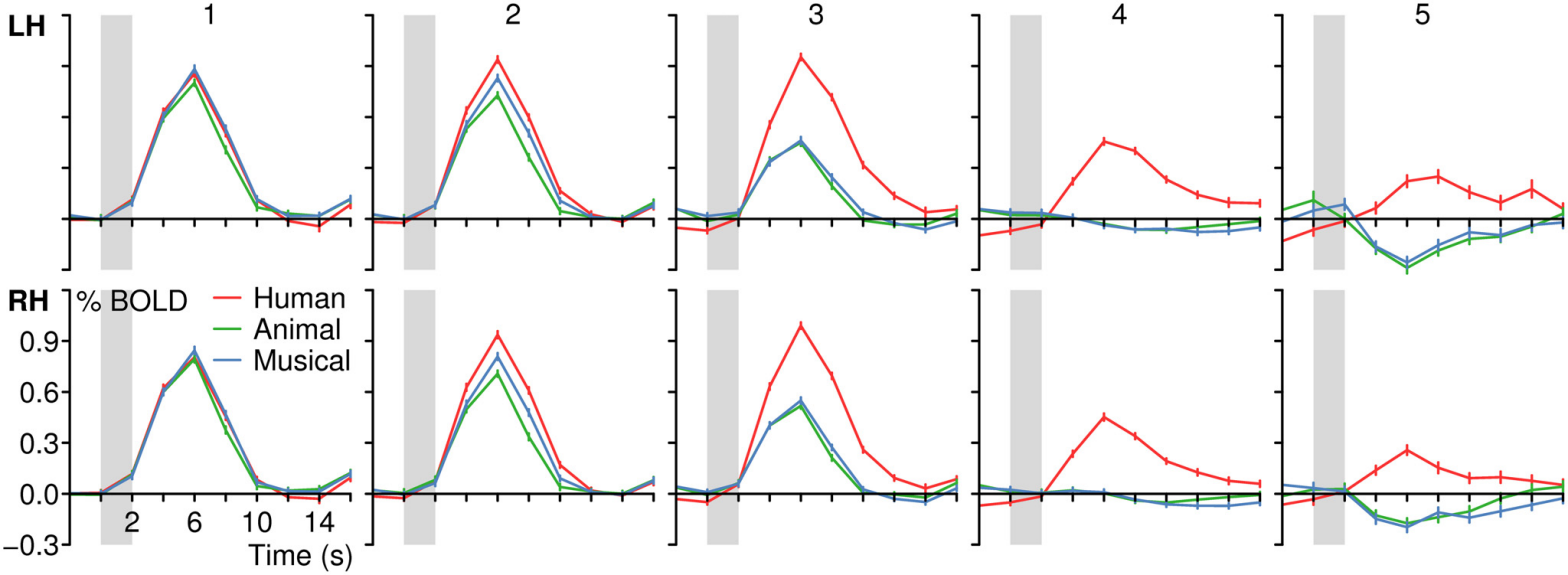
**BOLD signal time courses in different areas of the left (LH) and right (RH) temporal lobe (multi-subject analysis)**. For details on the origin of the different activation patterns 1–5 see Figure 5 or Table 3. The period of stimulus presentation is indicated by gray bars.

In a next step, each voxel that was significantly activated by human voices (H^+^) was analyzed for its specificity regarding voice processing (for details on the procedure see section Activation maps). It was found that voxels with similar specificity clustered together and did not scatter randomly over the temporal lobes. Therefore, five subregions with different activation patterns could be identified (1–5), each having a specific localization compared to the other subregions (Figure 5). The first region (pattern 1), which was located in the uppermost part of the STG, responded with similar BOLD intensity to all types of stimuli (Figure 6, Table 4). In the other areas, which were located increasingly more inferiorly and also slightly more anteriorly within the superior temporal lobes, the signal to human voices was significantly larger than to sounds of animals and musical instruments. The magnitude of the signal differences, however, rose gradually from the area with pattern 2 to the area with pattern 5. In area 2, only minor differences in the BOLD amplitudes of the voices and the other sounds could be observed. Area 3 presented a preferential activation pattern with clear differences in the BOLD amplitudes but significant activation both to human voices and the other sound categories. The areas 4 and 5 showed a selective activation pattern with significant activation to voices only. Concerning the latter two areas, the response to animals and musical instruments did not differ significantly from zero in area 4 (selectivity), but the non-vocal stimuli evoked a significant deactivation in area 5 (strong selectivity).

**Figure 7:**
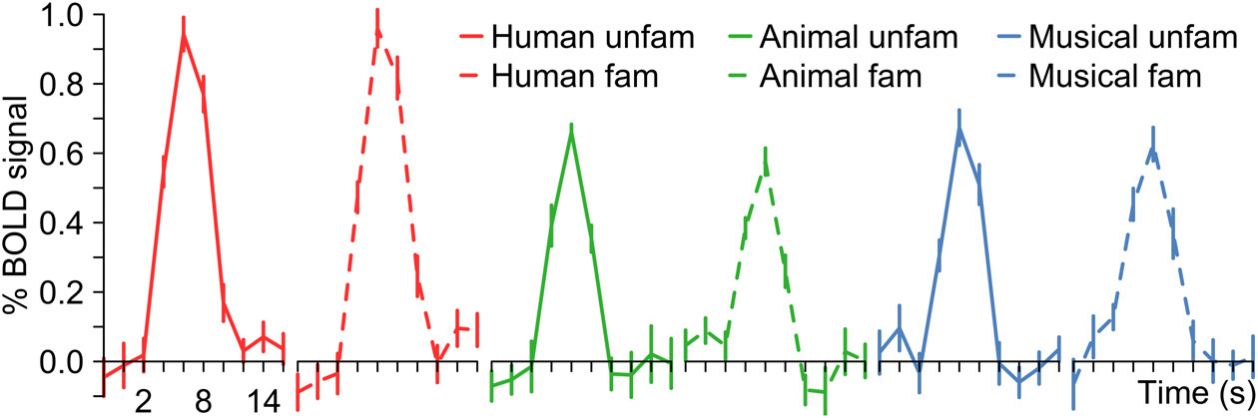
**BOLD signal time courses in the temporal lobes (combined left and right hemisphere) depending on stimulus category and the ability of the subjects to name the person, animal or instrument**.

Stimuli with and without name retrieval were analyzed collectively because the aim of the current study was to compare the neural response to the identification of three different types of acoustic categories in general. The combination was justified by the observation that the experimental design did not result in obvious within-category activation differences between named and non-named items (Figure 7).

Altogether, the data confirmed a deep involvement of the temporal lobes in a task that required the subjects to identify famous persons by their voices. Moreover, there were clues to the existence of functional subregions. Whereas more superior parts of the temporal cortices responded with similar BOLD intensities to human voices and to sounds of animals and musical instruments, the BOLD signal observed in areas above and below the STS showed a clear preference or even selectivity for human voices. For example, the distance between the centers of area 1 (similar BOLD intensity to all sound categories) and area 4 (selectivity) was about 20 mm (Table 4, see z coordinates). The cluster centers also differed with respect to their position along the y-axis of the brain (see y coordinates). The centers of the clusters in which the activation level differed highly between the voices and the other sounds (e.g., area 4) were located approximately 15 mm anterior to the activation centers with very similar BOLD intensities by all stimulus types (area 1). The area with the highest specificity regarding human voice processing was found bilaterally in the anterior part of the MTG.

#### 3.2.2. Separate-subject analysis using individually defined ROIs

To examine the observed differences between human voices and the other acoustic stimuli in further detail, the multi-subject analysis was extended by analyzing each subject's response pattern separately using individually defined regions of interest (ROIs). These were 12 ROIs in each hemisphere covering large parts of the left and right temporal lobe (see Figure 2). The extracted values (number of activated voxels, BOLD signal intensities, and selectivity indices) are given in Table 5. In most regions, significant activation (single-subject GLM, contrast H^+^ at *t* ≥ 4.0 corresponding to *q*_FDR_ < 0.001) was observed in all 12 participants. In three ROIs, significant activation was evoked by less than 12 subjects (*a-iSTG-LH*, 11 subjects; *a-sMTG-LH*, 9 subjects; *a-sMTG-RH*, 11 subjects).

**Table 5:** **Details for the regions of interest identified by the separate-subject analysis**.

		**a**		**ma**		**mp**		**p**	
		**LH**	**RH**	**LH**	**RH**	**LH**	**RH**	**LH**	**RH**
(a) Voxels	**sSTG**	646	685	1902	2080	3329	3135	1182	789
		175	180	190	135	163	172	179	214
	**iSTG**	549	563	2143	2392	3101	3184	1288	1283
		128	136	164	174	179	166	160	180
	**sMTG**	132	178	679	1097	963	2034	840	901
		38	67	145	149	164	219	160	205
(b) Human	**sSTG**	0.893	0.995	1.084	1.263	1.344	1.223	0.701	0.579
		0.102	0.060	0.087	0.116	0.082	0.085	0.049	0.058
	**iSTG**	0.676	0.768	0.806	0.925	1.007	0.938	0.784	0.738
		0.044	0.037	0.021	0.041	0.052	0.043	0.051	0.076
	**sMTG**	0.643	0.659	0.632	0.806	0.817	0.894	0.661	0.725
		0.054	0.060	0.043	0.047	0.059	0.049	0.035	0.081
(c) Animal	**sSTG**	0.369	0.528	0.739	0.850	1.127	1.060	0.640	0.605
		0.099	0.107	0.090	0.107	0.068	0.076	0.075	0.097
	**iSTG**	0.054	0.143	0.344	0.448	0.591	0.560	0.379	0.458
		0.049	0.071	0.053	0.051	0.057	0.051	0.068	0.086
	**sMTG**	−0.091	0.008	0.022	0.141	0.153	0.371	0.404	0.384
		0.059	0.059	0.038	0.057	0.071	0.057	0.053	0.071
(d) Musical	**sSTG**	0.564	0.734	0.959	1.046	1.289	1.181	0.727	0.662
		0.120	0.126	0.105	0.103	0.068	0.068	0.082	0.109
	**iSTG**	0.159	0.229	0.396	0.506	0.630	0.593	0.365	0.446
		0.057	0.072	0.044	0.045	0.065	0.055	0.072	0.113
	**sMTG**	−0.140	−0.007	0.027	0.146	0.155	0.394	0.462	0.399
		0.066	0.059	0.045	0.060	0.066	0.061	0.048	0.072

*(a) Number of voxels activated by human voices compared to rest (*H*^+^), (b–d) BOLD intensities. Given are the mean and the standard error of the mean. *a*, anterior; *iSTG*, inferior STG; *LH*, left hemisphere; *ma*, mid-anterior; *mp*, mid-posterior; *p*, posterior; *RH*, right hemisphere; *sMTG*, superior MTG; *sSTG*, superior STG*.

**Figure 8:**
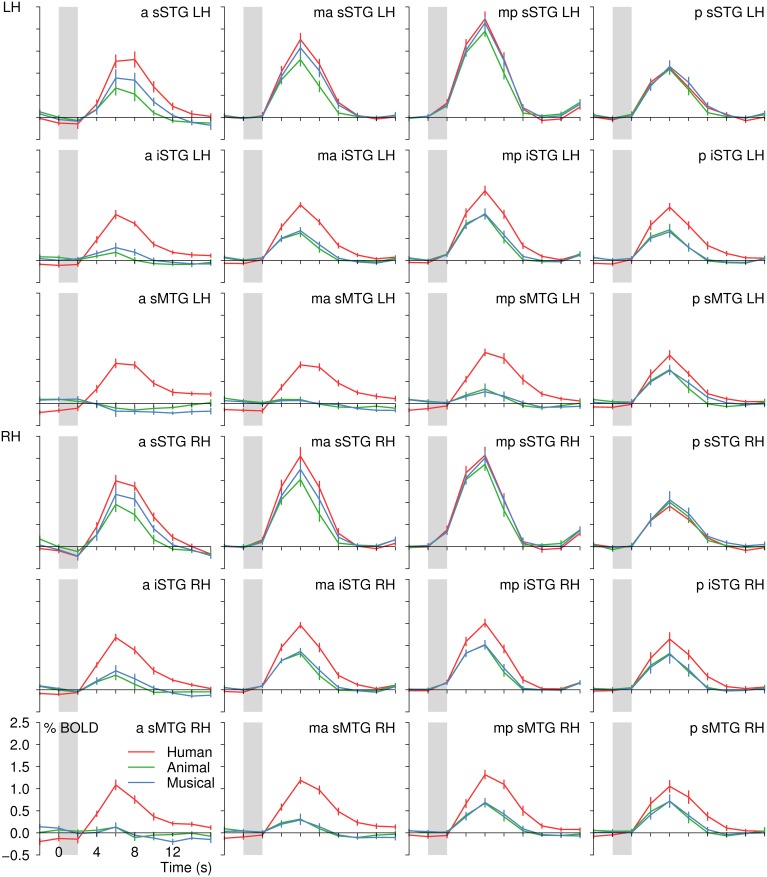
**BOLD signal time courses in all regions of interest of the left (LH) and right (RH) temporal lobe (separate-subject analysis)**. For details on the location of the regions see Figure 2 and Table 1. The period of stimulus presentation is indicated by gray bars.

Across each hemisphere, the separate-subject analysis confirmed the presence of differences in the activation level between the different stimulus types [*F*_(2)_ ≥ 76.2, *p* < 0.001; Figure 8]. In particular, human voices evoked higher signal intensities than sounds of animals or musical instruments [*F*_(1)_ ≥ 70.0, *p*_*B*_ < 0.001]. The latter two sound categories, however, did not differ significantly in their elicited BOLD signal [*F*_(1)_ ≤ 5.9, *p*_*B*_ ≥ 0.108]. Examining each ROI separately, activation differences between the three sound categories were observed in all regions [*F*_(2)_ ≥ 7.5, *p* ≤ 0.004] except for the ROIs termed *p-sSTG-LH* and *p-sSTG-RH* [*F*_(2)_ ≤ 1.6, *p* ≥ 0.227]. In most ROIs with significant differences between the conditions, human voices excited higher BOLD amplitudes than both animals and musical instruments [*t*_(11)_ ≥ 3.1, *p*_*B*_ ≤ 0.030]. In three ROIs (*ma-sSTG-LH, mp-sSTG-LH* and *mp-sSTG-RH*), the BOLD signal to human voices differed significantly from animal sounds [*t*_(11)_ ≥ 3.3, *p*_*B*_ ≤ 0.022] but not from musical instruments [*t*_(11)_ ≤ 1.4, *p*_*B*_ ≥ 0.597]. Altogether, ROIs that did not consistently show a preference for human voices were bilaterally located in more posterior parts of the *sSTG*.

Whereas the mid- to posterior *sSTG* did not reliably differentiate between human voices and sounds of animals and musical instruments, the anterior MTG distinguished most sharply between these conditions with the absolute BOLD signal difference between human voices and the control conditions being about 1.0%. In between, the BOLD signal differences in favor of the human voices increased gradually, which made the activation in the different parts of the temporal lobes resemble a processing stream from the auditory cortices to the anterior MTGs (Figure 8).

Moreover, the BOLD signal in the anterior MTG showed a strictly voice-selective activation pattern. Animal sounds and musical instruments did not evoke a significant activation in the ROIs *a-sMTG-RH* and *ma-sMTG-LH* [*t*_(11)_ ≤ 0.6, *p* ≥ 0.558] and even a slight, non-significant deactivation in ROI *a-sMTG-LH* [*t*_(11)_ ≥ −2.1, *p* ≥ 0.068]. Hence, the pattern seems to fulfill the criterion of voice selectivity in the strict sense with no significant activity evoked by any of the control conditions. As a more valid test for the absence of activation, the non-superiority (i.e., one-sided) version of the equivalence test was recruited. The test confirmed that the upper 97.5% confidence boundary of the BOLD signal evoked by animals and musical instruments in ROI *a-sMTG-LH* was within our defined equivalence limits of one tenth of the signal elicited by human voices, which means that the signal to the animals and musical instruments was significantly lower (*p* ≤ 0.025) than one tenth of the signal by the voices. This result statistically confirmed the absence of neural activity by the control conditions (also see section Statistics).

The variability in the magnitude of the signal differences in the temporal lobes was produced by systematic changes in the BOLD signal intensities. The BOLD signal of all sound categories was largest in the mid-posterior part of the *sSTG* and decreased toward more inferior and more anterior ROIs. Yet, the signal decrease was larger for sounds of animals and musical instruments than for human voices, with the result that the signal differences increased toward the anterior MTG (Figure 8). The statistical analyses revealed that the BOLD signal varied significantly both along the superior-inferior axis of the temporal lobes [*F*_(2)_ ≥ 50.9, *p* < 0.001] and along the posterior-anterior axis [*F*_(3)_ ≥ 9.3, *p* < 0.001]. *Post-hoc* analyses showed that there was a gradual BOLD signal decrease from the *sSTG* to the *iSTG* and again to the *sMTG* [*F*_(1)_ ≥ 31.6, *p*_*B*_ ≤ 0.001]. Significant BOLD signal decreases along the posterior-anterior axis were demonstrated from the *ma*- or *mp*-ROIs to the *a*-ROIs [*F*_(1)_ ≥ 28.3, *p*_*B*_ ≤ 0.004] and from the *mp*-ROIs to the *p*-ROIs [*F*_(1)_ ≥ 14.6, *p*_*B*_ ≤ 0.017]. More importantly, however, there was a significant interaction between the BOLD amplitude across the temporal lobes and the three sound categories [superior-inferior, *F*_(4)_ ≥ 25.4, *p* < 0.001; posterior-anterior, *F*_(6)_ ≥ 9.8, *p* < 0.001]. The signal decrease from the mid-posterior *sSTG* toward the anterior *sMTG* was more pronounced for animals and musical instruments than for human voices.

In summary, the analyses revealed that the specificity of the neural response in favor of the voices increased from the auditory cortices in the middle and posterior STG toward the MTG and the ATLs. In the anterior MTG, voice-selective activation was observed with significant neural activity to voices only. Animals and musical instruments either resulted in significant deactivation (multi-subject analysis) or in significant non-activation, which was confirmed using the approach of equivalence testing (separate-subject analysis). Hence, the most impressive finding of the current study was the gradual change in voice processing specificity from a comparable BOLD response to all stimulus types in the early auditory cortices to strict voice selectivity in the anterior MTG.

## 4. Discussion

### 4.1. Candidates for voice-selective areas in the strict sense

The aim of the present fMRI study was to assess the degree of voice-specific processing in the superior and middle temporal cortices when human voices were compared to sounds of animals and musical instruments. Consistent with other studies on voice processing (e.g., Belin et al., 2002; Fecteau et al., 2004), we found that the BOLD response in large parts of the temporal cortices was stronger to human voices than to the other environmental sounds.

Such findings were interpreted as evidence for the presence of voice-selective areas in the temporal lobes (Belin et al., 2000). Similarly, parts of the ventral temporal cortices have been claimed to serve as face-selective areas (Kanwisher et al., 1999). Those labels imply that these regions respond to voices or faces only, but not to other stimuli. However, these studies described a preferential rather than a selective pattern with significant activation also to control stimuli. Actually, selectivity was often explicitly defined in terms of preference, for example as “a greater neural activity” (Fecteau et al., 2004) or as a signal “at least twice as strongly to preferred as to non-preferred stimuli” (Spiridon and Kanwisher, 2002). In this vein, all areas in the current study could be termed selective for human voices except for the middle and posterior parts of the *sSTG*, which comprises large parts of the auditory cortex areas BA 41, 42, 22, and 52 (Brodmann, 1909).

However, more appropriate is to preserve the term *selectivity* for activation when not only the main condition evokes a stronger signal than control conditions but when it can also be statistically proven that the control conditions do not raise the activation level above a specific baseline. Otherwise, we suggest to use a term like *preference* (see also Joseph et al., 2006; Pernet et al., 2007). Moreover, since most attempts to detect selective activation aim at the identification of cognitive modules (Fodor, 1983), a preferential activation should result in the conclusion that the actual cognitive module has not yet been identified or that no such module is represented in the brain area under consideration.

The current study identified the anterior portions of the MTG as potential voice-selective areas. In these brain regions, control stimuli either produced no signal change (as was confirmed by equivalence testing) or a significant negative deflection of the BOLD response compared to the resting baseline. Hence, the only candidates for truly voice-selective areas in the temporal lobes were the anterior MTG but neither the upper bank of the STS (Belin et al., 2000) nor more posterior parts of the MTG (Fecteau et al., 2004). As far as the anterior MTG are concerned, our results do not speak against a cognitive module specialized for voice processing. But the results clearly rule out the upper bank of the STS and more posterior parts of the MTG as the sites for its representation, because of a strong response to other environmental sounds.

But even if selectivity is statistically confirmed, there is a need for caution for at least three reasons. (i) It is often difficult to match all but one stimulus feature. In the present study, human voices were compared with sounds of animals and musical instruments. These stimuli differ in various respects (see section 4.2), which prevents us from drawing firm conclusions about the nature of the potential cognitive module. (ii) An infinite number of comparisons is required. Our experiment did not prove the representation of a module in the anterior MTG because voices were compared to only two control conditions. As soon as a single proof of the contrary is given by one other stimulus, the hypothesis about the existence of a selective area is falsified. (iii) Finally, the choice of the baseline has effects on the activation level of control stimuli. Several lines of research argue for a generalized role of the ATLs in semantic processing including basic level items (see section 4.3). We suppose that an active baseline condition, which had prevented the subjects' from semantic processing during rest, has the capacity to demonstrated that also semantically simpler basic level items like animals and musical instruments activate the anterior MTG. Hence, the detection of null activation is closely tied to the choice of an appropriate baseline.

### 4.2. What stimulus characteristics caused the voice-specific activation?

The voice-selective activation in the anterior MTG may have several origins; one is speech recognition. Since spoken utterances carry information both about linguistic and vocal features, we cannot exclude that the preference for voices was influenced by linguistic processing (Scott et al., 2000; Davis and Johnsrude, 2003). However, evidence that the voices themselves made a substantial contribution comes from studies that presented non-speech vocalizations (Belin et al., 2002), that directly compared voice to speech recognition (Belin and Zatorre, 2003; von Kriegstein et al., 2003), or that revealed influences from voice familiarity (Birkett et al., 2007). All of them hinted at voice-related processing in the ATLs. Of particular importance in that respect is our own recent study (Bethmann et al., 2012), which compared familiar to unfamiliar voice processing using the same region of interest approach as in the current study. The same areas that revealed stronger responses to voices than to other environmental sounds in the present study were activated more by familiar than by unfamiliar voices in the previous experiment. In addition, in both studies a gradual increase in the signal difference between voices and other sounds (current experiment) and between highly familiar and unfamiliar voices (previous experiment) was found from the posterior STG to the anterior MTG. Commonly, the findings argue for a specific role of the temporal lobes in voice recognition. Moreover, the current study used a speaker identification task and thus focused on the voice rather than linguistic features of the utterances. As it was found that task demands exert influences on the BOLD signal (Brechmann and Scheich, 2005; Harinen et al., 2013), the preferential activation for voices was presumably effected by voice-related processes.

Second, the observed activation differences in the anterior MTG might stem from acoustic differences between the stimuli. Voices, for example, had significantly lower fundamental frequencies than the animal sounds and musical instruments. But we think that acoustic differences were not more than an additive component because the mid-posterior and posterior *sSTG*, which comprises large parts of classical auditory cortex areas (BA 41, 42, 22, and 52) (Brodmann, 1909; Brechmann et al., 2002); (Kaas and Hackett, 2008), did neither exhibit selective nor preferential activation. Furthermore, human voices ranged between animal and musical sounds when parameters like jitter, shimmer or acoustic-to-noise ratio were considered. If acoustic features had had a major impact on the BOLD signal, we had also seen activation differences between sounds of animals and musical instruments (Figure 8).

However, human voices differed from animal sounds and musical instruments in one crucial aspect. The fundamental frequencies varied significantly less than those of the other sounds, i.e., the voices were more similar to one another than were the other sounds. Closer perceptual similarity is usually associated with slower response times and lower recognition rates (Lloyd-Jones and Humphreys, 1997; Vigliocco et al., 2002). Accordingly, the response times were longer and the number of nameable items was lower for voices than for the control sounds. Hence, greater task demands represent a third reason for the stronger BOLD signal to voices. But what made human voices more similar to one another and their identification more difficult? In contrast to basic level items like animals and musical instruments, person recognition requires individual level semantic processing. Since semantic neighbors at the individual level (*Barack Obama, George W. Bush*) share more semantic and perceptual features than entities at more superordinate semantic levels (*cat, dog*), the discrimination and identification of individuals is more difficult. In the next section, we lay down our arguments why we think that subordinate level semantic processing is the most probable reason for the observed voice-selective activation in the anterior MTG.

### 4.3. The role of the anterior temporal lobes

Assuming that the ATLs are involved in voice processing, evidence suggests their participation in later speaker identification processes than in the acoustic analysis of vocal features. Imaging studies demonstrated a clear association of the ATLs with voice familiarity (Nakamura et al., 2001; Birkett et al., 2007; Bethmann et al., 2012) and lesion studies with deficits in familiar speaker identification despite preserved abilities in acoustic voice processing (Hailstone et al., 2010). For this reason and because a speaker identification task was used, we argue that post-acoustic aspects of voice recognition triggered the neural activity seen in the current investigation.

Yet, although we observed voice-selective activation, we are sure that the function of the ATLs goes beyond the process of voice processing. Not only speaker identification, but also familiar face and name recognition was found to evoke neural activity in the ATLs (Gorno-Tempini et al., 1998). This was especially true when biographical facts had to be encoded in addition to face, voice, or name information (Tsukiura et al., 2006; Joassin et al., 2011). The ATLs seem to be associated with the retrieval of biographical information about identified persons (Brambati et al., 2010). Accordingly, generalized person recognition deficits were observed to result from ATL lesions (Joubert et al., 2006; Gainotti, 2013).

Strict voice selectivity was seen in the anterior MTG but almost the whole temporal lobes responded more strongly to voices than to the other sounds. The signal difference was virtually absent only in the mid-posterior and posterior sSTG. In between, a gradient of increasing voice specificity appeared. Such a gradual pattern is in good agreement with previous hypotheses about a hierarchical stream to the ATLs necessary for the processing of vocalizations (Rauschecker and Scott, 2009), which was inspired by the concept of a “what” pathway in the visual domain (Mishkin et al., 1983). Along these streams, voice and face processing are assumed to become increasingly more abstract and independent of lower-level acoustic (Warren et al., 2006) or visual features (Nasr and Tootell, 2012). The additional finding that the ATLs differentiate both between familiar and unfamiliar voices and faces (see previous paragraphs) suggests that the processing streams are dedicated to the task of identifying persons by their voices or faces.

Other proposals hold that the ATLs are involved not only in person recognition but in the recognition of all types of unique or subordinate concepts (Rogers et al., 2006; Tranel, 2009). Again, a gradual pattern emerged across the temporal lobes. Tyler et al. (2004) showed that superordinate and subordinate level processing of visually presented objects activated the posterior part of the inferior temporal cortices. Toward the ATLs, the response to superordinate but not to subordinate level processing continuously decreased with the result that the signal difference in favor of subordinate processing increased. Very similar, our data revealed an increase in the signal difference between unique and basic level auditory concepts from the mid-posterior sSTG to the anterior sMTG. The striking similarity suggests that information from the sensory cortices is forwarded to the ATLs, which are increasingly less involved in superordinate level processing but continue to process specific and unique level concepts.

The convergence zone theory by Damasio (1989) is well suited to explain these results. It assumes that basic features of objects are distributively represented in early sensorimotor areas. The integration of these features to holistic concepts is achieved by multiple hierarchically organized stations, called convergence zones. One integration pathway is assumed to run from posterior to anterior temporal cortices. Regarding the present results, the model may be interpreted such that early convergence zones that process basic features are required by all types of concepts. Increasingly more complex concepts, however, will additionally involve increasingly more anterior regions. Hence, the theory predicts an increasing signal difference between unique/subordinate and more general items along the pathway to the ATLs.

Despite theoretical and empirical evidence for a differential involvement in specific and superordinate level semantic processing, our data are not incompatible with the assumption that the ATLs process several kinds and levels of concepts. This hypothesis is based on findings that ATL lesions cause generalized semantic deficits (Patterson et al., 2007). In contrast, imaging studies only seldom found the ATLs engaged in semantic processing. This failure was explained to result from resting or control conditions that were not sufficiently demanding to suppress semantic processes during these intervals (Price et al., 2005; Binder et al., 2008; Visser et al., 2010). In the present study, experimental and control conditions were of unequal difficulty regarding semantic retrieval and were compared with passive rest. During rest, semantic processing certainly continued. This produced a high baseline activity that was surpassed by the speaker identification task but not by the simpler identification of animals or musical instruments.

Accordingly, the ATLs were identified as parts of the default mode network, whose activity decreases during active tasks and increases during periods of passive fixation (Binder, 2012). In our study, animal and instrument identification produced a slight deactivation in the anterior MTG compared to the resting baseline. Yet, instead of assuming that semantic processing stopped during these periods, we suggest that the task was less demanding than spontaneous semantic processing during rest. We agree with Binder (2012) that “task-induced deactivation should not occur, or should be much weaker, when the explicit task engages the same processes that are engaged during rest.” In line with this, the signal decrease in the ATLs during semantic tasks was smaller than during phonological or perceptual tasks (Wirth et al., 2011), indicating that these structures are involved in semantic processes both during periods of rest and during active semantic tasks. Strong signal increases, however, will only be observed with demanding semantic tasks such as person identification.

## Author contributions

Substantial contributions to the conception or design of the work: Anja Bethmann and André Brechmann. Performed the experiments, analyzed the data and contributed analysis tools: Anja Bethmann. Drafting the work or revising it critically for important intellectual content: Anja Bethmann and André Brechmann. Final approval of the version to be published: Anja Bethmann and André Brechmann. Agreement to be accountable for all aspects of the work in ensuring that questions related to the accuracy or integrity of any part of the work are appropriately investigated and resolved: Anja Bethmann and André Brechmann.

## Funding

This work was supported by the German Research Foundation grants “Active Auditory System” SFB/TRR 31 (http://www.dfg.de/en/research_funding/programmes/list/projectdetails/index.jsp?id=14945932) and “Companion Technology” SFB/TRR 62 (http://www.dfg.de/foerderung/programme/listen/projektdetails/index.jsp?id=54371073).

### Conflict of interest statement

The authors declare that the research was conducted in the absence of any commercial or financial relationships that could be construed as a potential conflict of interest.
